# Bilateral Persistent Hyperplastic Primary Vitreous: A Case Report and Review of the Literature

**DOI:** 10.7759/cureus.13105

**Published:** 2021-02-03

**Authors:** Hamza Maqsood, Shifa Younus, Maham Fatima, Muhammad Saim, Shaheryar Qazi

**Affiliations:** 1 Cardiology, Nishtar Medical University, Multan, PAK; 2 Medicine, Nishtar Medical University, Multan, PAK; 3 Radiology, Nishtar Medical University, Multan, PAK

**Keywords:** phpv, leukocoria, retinoblastoma, ophthalmology, vitreous

## Abstract

Bilateral persistent hyperplastic primary vitreous (PHPV) is a rare ocular disorder. Its clinical manifestations include bilateral corneal haziness, microphthalmia, and cataract. It is the second most common cause of leukocoria after retinoblastoma. Most cases of PHPV are unilateral. The typical imaging features of PHPV comprise bilateral echogenic masses and a fibrous cord extending from the posterior surface of the lens to the optic disc. In this report, we present a case of bilateral PHPV in an infant who presented with bilateral corneal haziness and watery discharge. A detailed ocular examination and knowledge about its features on imaging can lead to a timely and accurate diagnosis of the condition.

## Introduction

Bilateral persistent hyperplastic primary vitreous (PHPV) is a rare vitreoretinal disorder. The most common clinical manifestations of the condition are leukocoria, microphthalmia, and cataract [1]. It is a congenital disorder of the eye, which occurs due to abnormal persistence of fetal intraocular vessels and embryonic vitreous [2]. Primary vitreous forms around the seventh week of fetal life and starts regressing in the 20th week. It is replaced by avascular secondary vitreous by birth. Failure of regression of primary vitreous can lead to PHPV [3]. As compared to unilateral PHPV, bilateral PHPV is rare and sporadic.

PHPV is one of the most important differential diagnoses of retinoblastoma [1]. It may present a diagnostic challenge for ophthalmologists and pediatricians. A detailed clinical examination along with radiological investigations is required for the diagnosis and efficient management of the disease.

## Case presentation

Our patient was a 20-day-old female infant who presented to an ophthalmology clinic with complaints of bilateral corneal haziness and watery discharge. The patient had been born at term by uneventful spontaneous vaginal delivery. There was no antenatal and family history related to presenting complaints. Systemic examination was normal. On ophthalmological examination, pupillary reactions were found to be normal. Bilateral corneal haziness was present. Bilateral pupillary reflexes were positive, and the size of the left cornea was relatively smaller than that of the right one. On slit-lamp examination, posterior sub-capsular opacification was observed along with a fibro-vascular thread-like extension from the posterior surface of the lens. At this stage, we were suspecting retinoblastoma.

**Figure 1:**
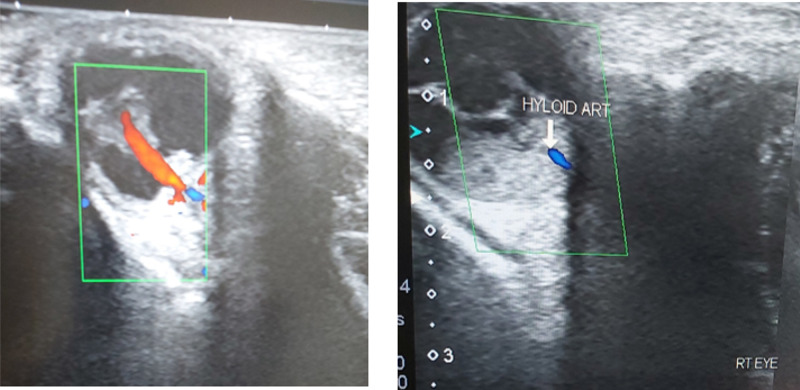
Doppler ultrasonography showing the presence of the hyaloid vasculature

We referred the patient to the department of radiology for further evaluation. The radiologist performed an ultrasound B scan for the identification of the pathology. Ultrasound showed echogenic masses in the posterior segment that were extending from the posterior surface of the lens to the optic disc. Doppler study showed the presence of hyaloid vasculature (Figure 1).

**Figure 2:**
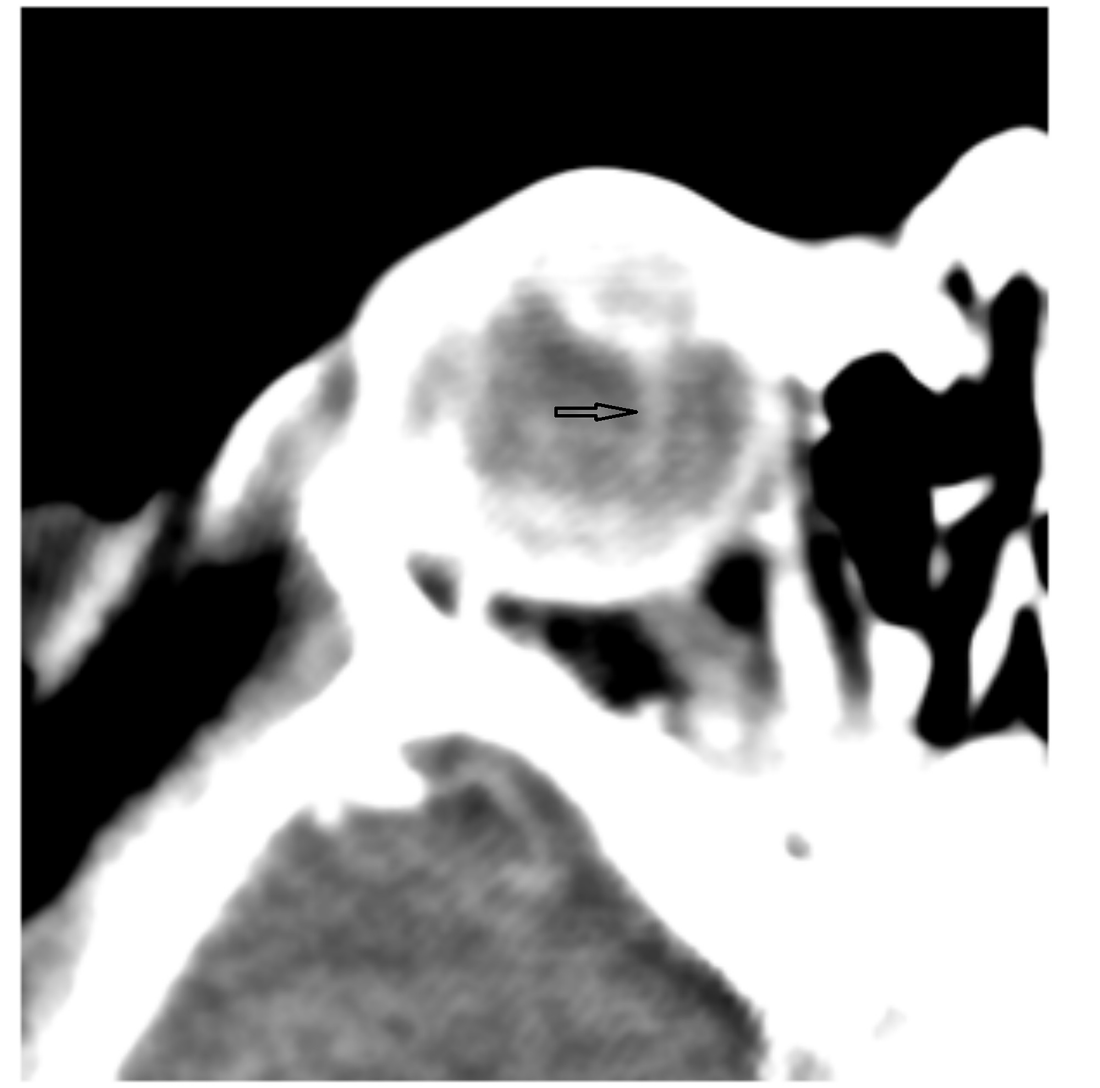
Post-contrast CT scan of the right orbit showing a thread-like extension (black arrow) in the posterior segment of the globe CT: computed tomography

**Figure 3:**
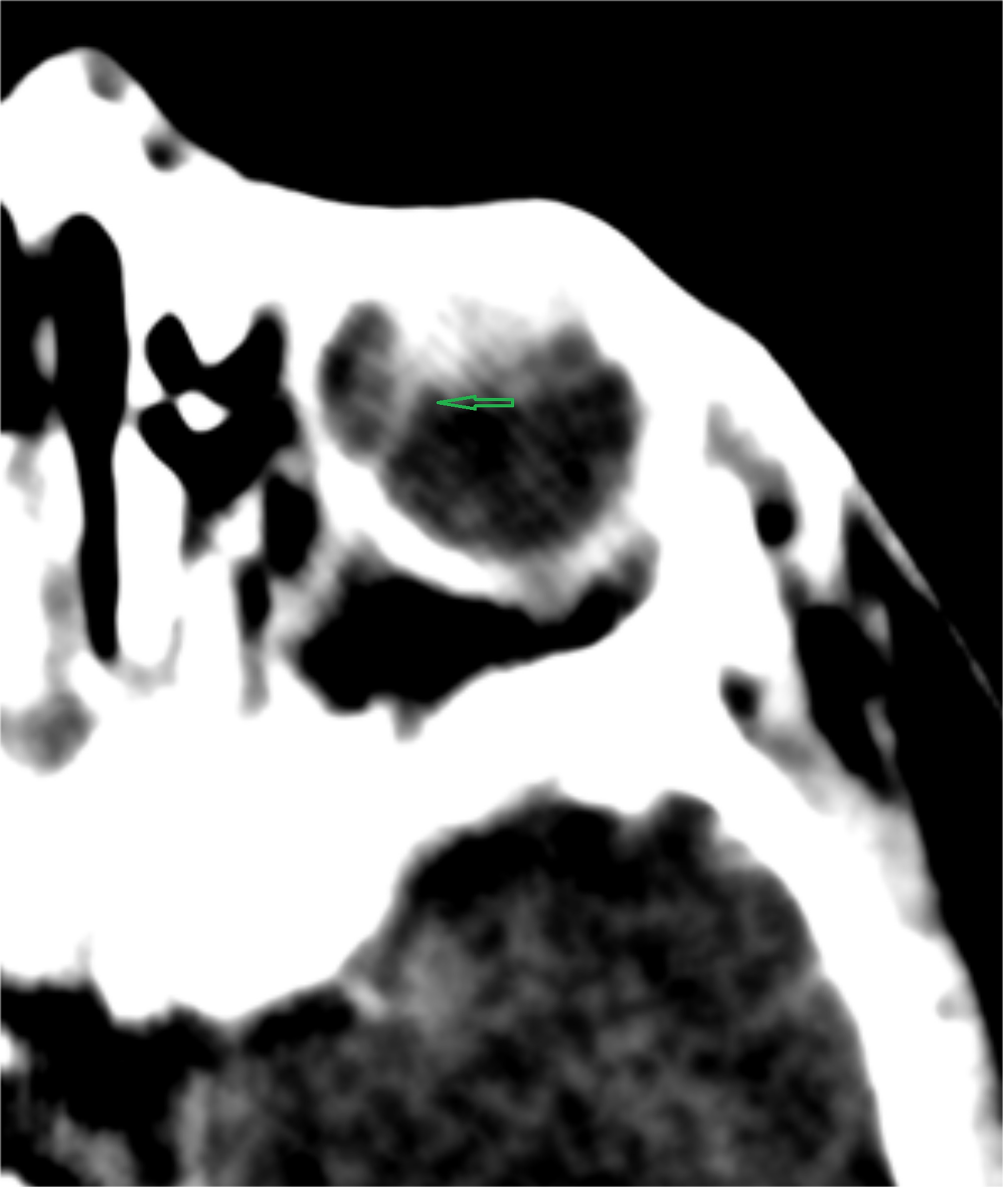
Post-contrast CT scan of the left orbit showing a similar thread-like extension (green arrow) in the posterior segment of the globe CT: computed tomography

**Figure 4:**
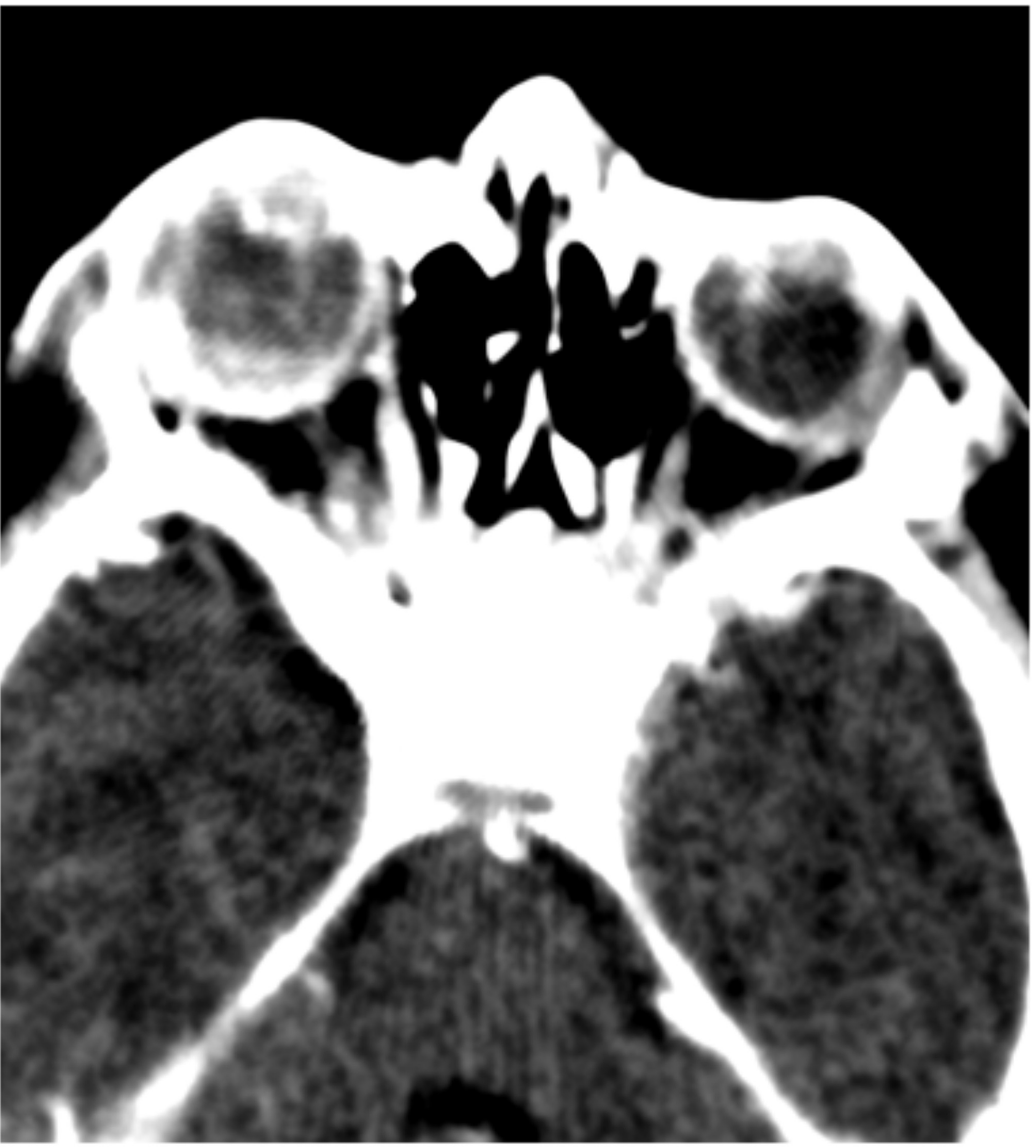
Post-contrast CT scan of both orbits showing a posterolental mass along with a thread-like extension in the posterior segment of the globes CT: computed tomography

We also observed reduced axial length of both eyes. The radiologist performed contrast CT scans. CT scans revealed posterolental masses along with a hyper-dense thread-like extension in the posterior segment of both eyes. It was representing hyaloid arteries. We did not observe any intra-lesion calcification (Figure 2, Figure 3, Figure 4).

After correlating the clinical presentation and imaging findings, we made a diagnosis of bilateral PHPV. The ophthalmologist performed an anterior capsulotomy with lens aspiration. A posterior capsulotomy was also done to remove the central plaque. For the removal of primary vitreous and persistent hyaloid vasculature, an anterior vitrectomy was performed. There were no intra-operative or postoperative complications. We followed up with the patient after four weeks. There was no ocular abnormality.

## Discussion

We reported a case of bilateral PHPV in a 20-day-old infant. She had no familial, antenatal, or postnatal history related to the disease. There was no history of trauma as well.

As embryonic development progresses, the primary vitreous and hyaloid vasculature are replaced by avascular secondary vitreous. This progressive regression starts in the 20th week of gestation and completes at birth [3]. Rarely, this regression fails and leads to the persistence of primary vitreous and hyaloid vasculature. PHPV is usually unilateral. Bilateral cases are rare and are mostly associated with certain other congenital disorders like Norrie disease, trisomy 13, 15, and 18 [2]. A study performed by Pollard revealed that the bilateral subtype of PHPV accounts for only 2.4% of all such cases [4].

The most common clinical signs and symptoms of PHPV include bilateral corneal haziness with watery discharge, microphthalmia, and decreased vision [5]. Based on the involvement of the ocular segment, PHPV is classified into three types: anterior, posterior, and mixed [1]. The posterior form is rare and accounts for only 22% of all cases whereas the anterior and the mixed form make up 36% and 42% of all cases respectively [6]. According to this classification, our case fell under the anterior type of bilateral PHPV.

Ocular ultrasound and CT scan are the two gold standard imaging modalities that have proven to be very significant in the diagnosis of PHPV. Ocular ultrasound reveals an echogenic cord extending from the posterior surface of the lens to the optic disc. It also shows the decreased axial length of the globe. Ocular ultrasound can also help in ruling out any calcification [7]. CT scan can aid in better visualization of the underlying pathologies and provisional diagnosis of PHPV. Although CT scan can better demonstrate all the findings of ultrasonography, it also carries the risk of radiation exposure.

In a patient with bilateral leukocoria, the differential diagnosis includes retinoblastoma, PHPV, retinopathy of prematurity, vitreoretinal dysplasia, and ocular toxocariasis [8]. Imaging modalities can help in excluding the diagnosis of retinoblastoma. Retinopathy of prematurity can be excluded by asking for detailed medical history and by supplementation of oxygen. All newborns presenting with bilateral leukocoria should be screened for congenital disorders like trisomy 13, 15, and 18 [8].

The management of PHPV depends on the age of the patient at the time of diagnosis and extension of the pathology. Mild cases can be managed conservatively due to their benign nature. Moderate and severe cases that are diagnosed late require surgical interventions. These interventions include anterior and posterior capsulotomy along with anterior vitrectomy [9].

## Conclusions

Bilateral PHPV is the second most common cause of leukocoria after retinoblastoma. Detailed ocular examination along with imaging can help in the early diagnosis of the disease. Prompt recognition, along with surgical intervention in severe cases constitutes the key to a successful outcome.
